# Microstructure Refinement via Nucleation of Intragranular Acicular Ferrite Stimulated by Ti-Containing Core–Shell Structured Particles in Low-Carbon Steel

**DOI:** 10.3390/ma17184644

**Published:** 2024-09-21

**Authors:** Zhu Yan, Chao Wang, Hua Duan, Junjie Hao, Guo Yuan

**Affiliations:** The State Key Laboratory of Rolling and Automation (RAL), Northeastern University, Shenyang 110819, China

**Keywords:** impact toughness, acicular ferrite, interfacial energy, Mn-depleted zone

## Abstract

This study investigated the microstructure, mechanical properties, and nucleation mechanism of acicular ferrite (AF) present in hot-rolled Ti deoxidized steel. In our experiments, the impact toughness of Ti deoxidized steel is significantly increased to 144 J at −20 °C, while those Mn and Al deoxidized steels are only 9 J and 18 J, respectively. Interlocked AF is the primary microstructure of Ti deoxidized steel. The second-phase particles of the core–shell-type structure, in which Ti_2_O_3_ is the nucleus and TiO is the outermost shell, act as effective nucleating agents to stimulate AF nucleation. The low lattice disregistry between TiO and AF is the main factor contributing to the production of AF. It is also revealed that Ti_2_O_3_ and MnS fulfill the particular orientation relationship, contributing to the formation of an Mn-depleted zone (MDZ) adjacent to MnS, proposed to be one of the possible mechanisms for promoting AF nucleation.

## 1. Introduction

The thermo-mechanical control process (TMCP) is commonly utilized for manufacturing and processing metallic materials because it considerably improves the overall mechanical qualities of steel. The microstructure refinement of hot-rolled steel achieved during the TMCP is obtained by controlling rolling and cooling. However, for steel plates with large final thicknesses, the impacts of controlled rolling and cooling are not evenly disseminated across the entire thickness of the sheet due to the capacity limitations of the rolling equipment and the differences in cooling rates between the sections, generating distinct transformation products at the center and on the surface, which can adversely affect the final properties. In addition, for a hot-rolled steel pipe with shaped section characteristics, the lack of direct regulation of controlled rolling under high-temperature deformation conditions means that the austenite grain size prior to transformation cannot achieve the desired conditions.

The introduction of a large number of finely dispersed distributions of special types of second-phase particles in steel not only refines the grain by pinning austenite grain boundaries at high temperatures but also acts as an ideal site for the heterogeneous nucleation of intragranular acicular ferrite (IAF), known as particle-stimulated nucleation (PSN) [1]. Then, the formation of the interlocking acicular microstructure deflects or terminates crack extension to enhance impact toughness [2,3,4]; this approach has been successfully applied in the field of welding.

During continued cooling, the austenite grain boundaries and nucleation sites inside the grains undergo a competitive transformation, ultimately creating AF [5]. Numerous investigations have demonstrated that the transition mechanisms of AF and bainite are comparable, as they preserve similar orientation relationships with the parent austenite and a diffusion-free shear mechanism plays a major role; therefore, these processes are considered to be essentially intragranular nucleated bainites [6,7]. Relying on the alloy content and particle characteristics, the balance of nucleation is shifted from the austenite grain to the intragranular regions above a critical austenite grain size. The nucleation balance of ferrite shifts from the austenite grain boundaries to the intragranular locations when the austenite grain size exceeds a critical value [8]. Valid ferrite nucleating particles and highly soluble boron inhibit ferrite nucleation at the grain boundaries [9,10]. Nevertheless, the most important issue is the existence of efficient second-phase particles. Effective particles that act as nuclei for highly ductile AF microstructures have lower activation energy barriers for ferrite production, and if there are no potent nucleating agents, AF presumably does not form [11].

Numerous studies have shown that some specific phases, especially Ti-rich phases, are often effective nucleating agents for AF, e.g., titanium nitride, ilmenite, and spinel. The mechanisms for the excitation of AF nucleation by Ti-containing particles include the following: (1) Low lattice mismatch degree theory. There is an epitaxial relationship between particles (TiN, TiC, TiO, MnTi_2_O_4_, etc.) and ferrite, which favors the precipitation of ferrite nuclei on the particle surface [12,13]. (2) Local austenite-stabilizing solute depletion zones theory. The AF-nucleation-promoting roles of localized manganese depletion zones (MDZs) around particles (Ti_2_O_3_, MnTiO_3_) are recognized by many researchers [14,15,16]. However, the origin of MDZs is still debated. Another version of this theory is the depleted carbon zone mechanism, through which the effectiveness of particles (TiO_2_) stems from the release of oxygen to decarbonize the adjacent steel matrix to produce a depleted carbon zone [17]; however, there are insufficient, direct experimental data to confirm the presence of carbon depletion around the particles.

Most studies of AF have been conducted on weld metals. The well-developed AF microstructures in steel weld deposits occur due to high particle density, assuring a high density of nucleation sites. In contrast, second-phase particle-induced ferrite nucleation in high-temperature hot-rolled low-carbon steels has rarely been studied. It is critical to have a clear understanding of which second-phase particles, which typically have inhomogeneous properties, are more effective for performing intragranular nucleation, as well as how such particles promote intragranular nucleation. This paper employed Ti-oxide particles to enhance the nucleation of intragranular ferrite in low-carbon steel, and this type of particle was found to be an efficient site for AF nucleation. The characteristics of Ti-oxide particles and their microstructural impacts were investigated, and the intragranular nucleation mechanism used was elucidated.

## 2. Experimental Materials and Methods

Three experimental steel plates (labeled MD, AD, and TD) were prepared using a 50 kg vacuum induction melting furnace. The chemical composition of the experimental steel is listed in Table 1. Ti deoxidized TD steel to obtain efficient Ti-oxide particles. MD and AD steels were deoxidized using Mn and Al, respectively, for comparison. The ingots were reheated to 1523 K (1250 °C) and held for 1 h before being rolled into 13 mm thick plates at a final rolling temperature of approximately 1323 K (1050 °C). The heated plates were subsequently cooled to 923 K (650 °C) at a rate of 15 °C/s by using a water jetting device, followed by air cooling to ambient temperature.

The microstructures of the specimens obtained after erosion using 4 vol% nitric acid alcohol solution were observed using an Olympus optical microscope (OM, Olympus, Tokyo, Japan). The morphologies and types of inclusions were analyzed using a Thermo Scientific Apreo 2C scanning electron microscope (SEM, Thermo Scientific, Waltham, MA, USA), while SYMMETRY S2 model electron backscatter diffraction (EBSD, Oxford, UK) was used to analyze grain boundary features with a scanning step of 1.3 µm. Specimens were prepared using mechanical polishing and argon ion polishing.

The chemical properties of typical second-phase particles were characterized using scanning transmission electron microscopy (STEM, FEI Talos F200X G2, Thermo Scientific, USA) after the ultrathin sectioning of the Ti oxide particle/matrix interface in TD steel using the CROSS BEAM 540 focused ion beam (FIB, Zeiss, Oberkochen, Germany) technique. To further investigate the AF nucleation mechanism, high-resolution electron microscopy (HRTEM) and selected area electron diffraction (SAED) techniques were utilized to study the crystallographic structure of the particle/matrix interface.

**Figure 1:**
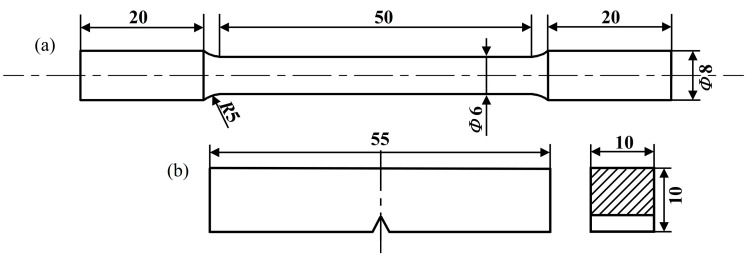
Dimensions of specimens for tensile (**a**) and impact tests (**b**).

Longitudinal tensile specimens, of 8 mm in diameter and 40 mm in gauge length, were intercepted from hot-rolled sheets in the rolling direction; the specific dimensional information for this procedure is shown in Figure 1. The tests were carried out using a CMT5105 tensile testing machine (MTS, Norwood, MA, USA) at a tensile speed of 3 mm/min, and the experimental temperature was set at room temperature. The low-temperature impact toughness of the specimens at −20 °C was examined by using an Instron 9250 HV drop weight tester(Instron, Norwood, MA, USA) to perform impact tests according to ASTM E23 standards [18], with specimens being cut parallel to the rolling direction and quasi-Charpy V-notch impact specimens with dimensions of 10 mm × 10 mm × 55 mm. The FEI QUANTA 600 scanning electron microscope (SEM, Thermo Scientific, USA) was used to observe the impact fracture morphology. Three specimens of each alloy were tested via mechanical properties experiments to ensure the reproducibility of the data.

## 3. Results

### 3.1. Microstructure of Hot-Rolled Steel Plates and Particles Characteristics

**Figure 2:**
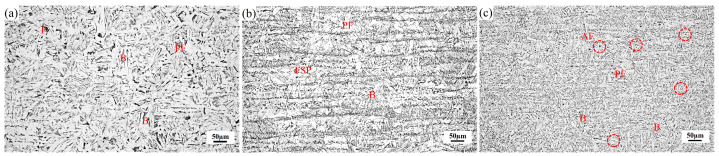
Optimal microscopy images of (**a**) MD steel, (**b**) AD steel, and (**c**) TD steel.

Figure 2 illustrates the optical micrographs of hot-rolled plates created using our experiment. We can see that coarse bainite (B), polygonal ferrite (PF), and a small quantity of pearlite (P) between the B plates constitute the microstructure of MD steel. PF, the ferrite side plate (FSP), and B dominated the microstructure of AD steel. The microstructure of TD steel was predominantly composed of AF, PF, and B, along with small amounts of P. Compared to MD and AD steels, TD steel had a greatly refined microstructure. In the meantime, the red dashed circles in Figure 2c represent the second-phase particles that act as AF nucleating agents in MD steel.

**Figure 3:**
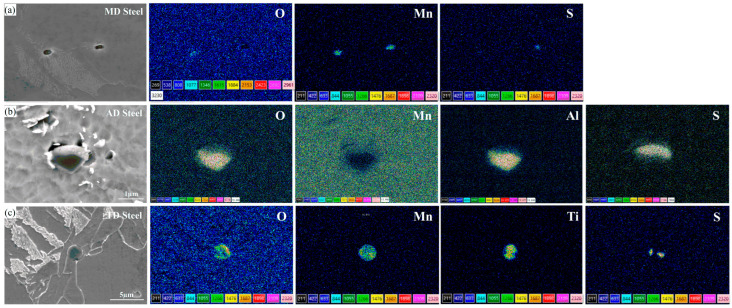
EPMA analysis of typical particles in (**a**) MD steel, (**b**) AD steel, and (**c**) TD steel.

Figure 3 depicts the morphologies and elemental mappings of typical second-phase particles in the three steels. The particles in MD steel were primarily MnO·MnS and MnS. In one study, J.H. Shim examined the roles of many particular phases on the nucleation of IGF, of which MnS singularly and MnO·MnS composite particles were shown to be inert [13], supporting the results shown in Figure 2a. Polygonal Al_2_O_3_·MnS particles were the main second-phase particles present in AD steel. Similar to the Ti-rich phase, the Al_2_O_3_ phase has garnered a great deal of scholarly interest. Dowling et al. suggested that Al_2_O_3_ would be one of the most effective nucleating agents due to its high surface energy [19]. It has also been reported that Al_2_O_3_ is only efficient when combined with other agents, such as Al_2_O_3_·MnO [8]. Furthermore, Yang [20] proposed that IGF might be nucleated at the complex phase made up of Al_2_O_3_ and MnS, which functions as an inert surface for straightforward heterogeneous nucleation. Based on their findings, equiaxed ferrite formed on the particles. Conversely, Li [21] and Liu [22] et al. showed that when no additional precipitates were present during the Al_2_O_3_·MnS composite phase, this compound was generally not an effective nucleant for AF. As shown in Figure 3c, the second-phase particles in TD steel are mainly subcircular Ti-O-Mn·MnS, and many AF plates grew at the edges of the particles. Thus, the Ti-O-Mn·MnS particles in TD steel can act as effective nucleation sites for AF.

### 3.2. Mechanical Properties of the Experimental Steels

**Figure 4:**
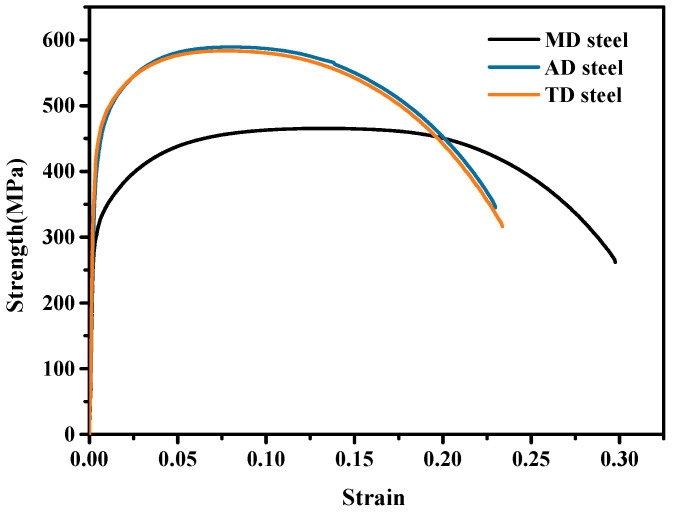
Stress–strain curves of the MD, AD, and TD steels.

Figure 4 illustrates the stress–strain curves of the experimental steel, as well as the results of the tensile tests (Table 2). All experimental steels exhibited continuous yield. AD and TD steels possessed similar tensile properties, having higher yields and tensile strengths than MD steel. Additionally, Table 2 depicts the impact toughness of the three steels at −20 °C. The impact toughness values of MD and TD steels were 18 J and 144 J, respectively, whereas that of AD steel was only 9 J.

### 3.3. Fracture Morphologies of the Experimental Steels

**Figure 5:**
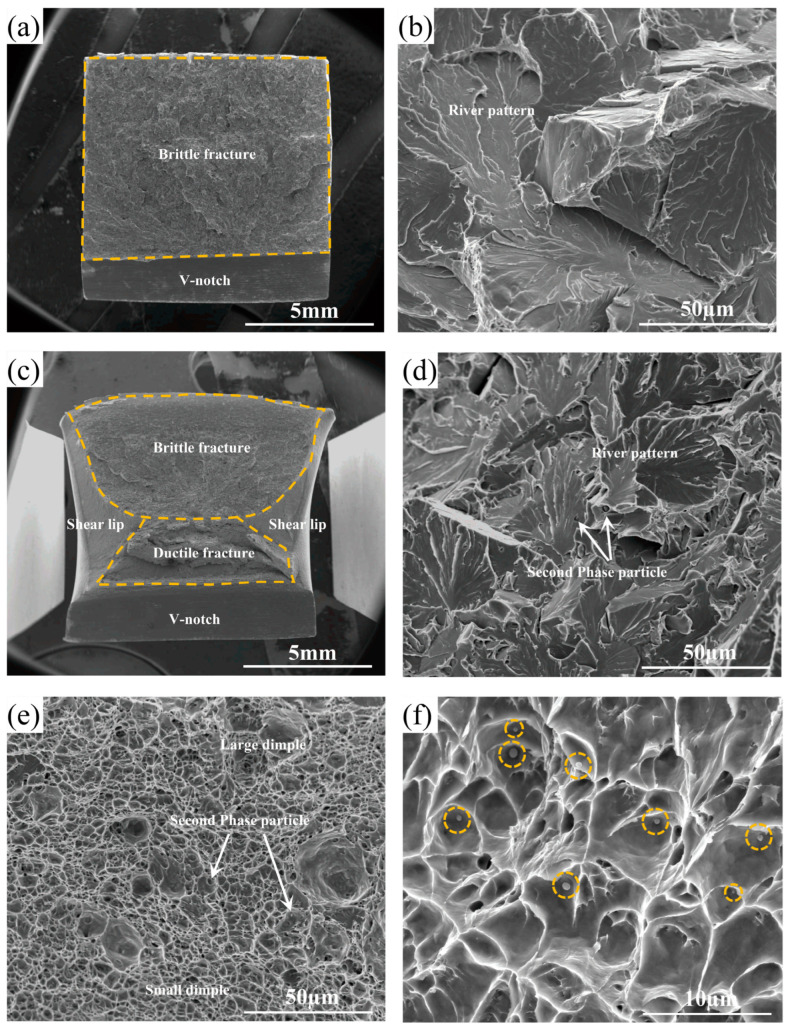
SEM images of (**a**,**b**) AD steel and (**c**,**f**) TD steel at −20 °C impact specimen fracture. (**a**) complete fracture cross-section of AD steel, (**b**) cleavage facet and coarse river pattern at the fracture of AD steel, (**c**) complete fracture cross-section of TD steel, (**d**) cleavage facet and coarse river pattern in the brittle fracture region of TD steel, (**e**) dimples of different sizes in the ductile fracture region of TD steel, and (**f**) second-phase particles present in the dimples.

Figure 5 depicts the SEM fractographs of AD and TD steels. The impact fracture of AD steel was characterized by typical brittle cleavage with no toughness zone. The fracture surface showed a discernible straight tearing ridge, cleavage facet, and coarse river pattern. In the meantime, Figure 5c illustrates that the impact morphology of TD steel exhibited a dual fracture pattern of ductile fracture and brittle fracture, where the fracture surface covered the ductile zone, the brittle zone, and the shear lip. Compared to AD steel, the size of the river pattern was reduced in TD steel, beneficial for toughness. Different-sized dimples formed within the toughness zone, and second-phase particles were also observed at the bottom of the dimples, as seen in the orange circle in Figure 5f, indicating that the formation of dimples was associated with fine second-phase particles. Some second-phase particles were also distributed within the brittle zone, but they were not the point of origin of the river pattern.

## 4. Discussion

### 4.1. Interaction between Microstructure and Toughness

**Figure 6:**
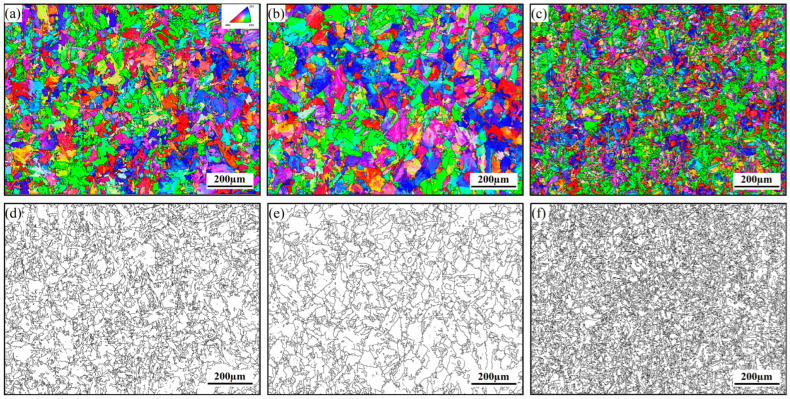
EBSD micrographs of (**a**,**d**) MD steel, (**b**,**e**) AD steel, and (**c**,**f**) TD steel, as well as (**a**–**c**) inverse pole figure maps, (**d**–**f**) grain boundary maps.

As shown in Figure 2c, the Ti-O-Mn·MnS second-phase particles were effective nucleating agents for AF, and the induced formation of a dominant AF structure increased the corresponding toughness values. The phase transformations of nucleation in the intragranular nucleation sites and at the grain boundaries are in a competitive process, and the preferred-phase transformation products would have a hindering effect on the later-phase transformation products. A specific density of effective second-phase particles provides sites for intragranular nucleation when the austenite grain size is known, and the nucleation equilibrium favors intragranular nucleation sites, resulting in favorable conditions for AF nucleation on the particle surfaces. As depicted in Figure 6, the AF plates effectively split the austenite grains while limiting the development of bainite packets, ultimately refining the microstructure. The average effective grain size (AEGS) was measured by EBSD, measuring 15.24 μm, 24.74 μm, and 11.44 μm for MD, AD, and TD steels, respectively. The AEGS corresponds to the size of the cleavage facets, which is a key structural unit affecting strength and toughness [23]. Prior observations of TD steel (Figure 5) revealed a huge number of dimples and small-sized cleavage facets, indicating that the production of AF greatly refined the AEGS, thus improving the impact toughness.

**Figure 7:**
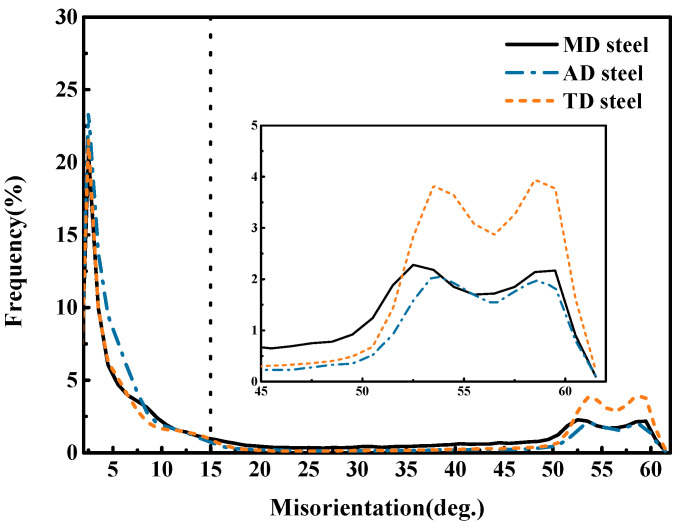
Grain boundary misorientation distribution of experimental steels.

On the other hand, the interlocking structure of “needle-like” ferrite provides randomly varying grain orientation, deflecting the crack extension path and contributing to the impact absorption energy. The results of this analysis demonstrate that the low-angle grain boundaries (LAGBs) separate adjacent bainite packets (Figure 6a–c), while ferrite laths inside the same bainite packets are all the same color. Since the AF plates and surrounding bainite typically have distinct Bain groups, the grain boundaries between them are typically high-angle grain boundaries (HAGBs). Obviously, the grain borders between bainite, polygonal ferrite, and AF are typically HAGBs larger than 15° (Figure 6d–f). Generally, microstructures with high proportions of HAGBs exhibit higher impact toughness. The existence of AF in TD steel led to a significant number of HAGBs being in the microstructure, and the refinement of the bainite packets further increased the proportion of HAGBs, beneficial for the suppression of crack propagation, as shown in Figure 7; as a result, the TD steel exhibited high toughness.

### 4.2. Mechanism of AF Nucleation Stimulated by Ti-Containing Particles

To explain the role of second-phase particles in carbon steel in inducing the nucleation of IAF, four possible mechanisms have been proposed, nucleation by localized solute depletion, nucleation at low-mismatch interfaces, nucleation in regions of high volumetric strain, and nucleation on energetic inert substrates based on the classical theory of heterogeneous nucleation.

The driving mechanism for AF nucleation is volumetric strain caused by variations in thermal expansion coefficients at the interface between the second-phase particles and the matrix. Barritte et al. [24] discovered that the idea that the nucleation of AF in the strain field around the particles stemmed from lattice distortion caused by the significant difference in thermal expansion coefficients between parts of the particles and the matrix was not supported by actual experimental results. Furthermore, the multi-phase nature of the second-phase particles made it more difficult to calculate the value of the volume change. The then-prevailing viewpoint relied on data from the coefficients of the thermal expansion of all phases in the particles to conduct theoretical calculations. Compared to the austenite-to-ferrite transformation volume free energy term, the strain energy resulting from variations in thermal expansion coefficients is typically insufficient to provide all the free energy required for ferrite nucleation [25,26]. Thus, it is doubtful that volumetric strain is the deciding factor in ferrite nucleation, and further discussion of the likelihood of nucleation in the strain energy field will be avoided.

It has also been suggested that these particles function as an inert substrate to lower the nucleation energy barrier and enable the nucleation of AF, according to the classical theory of heterogeneous nucleation [27]. Currently, the primary determinants involved in the development of AF are the quantity and size of the particles. Meanwhile, the application of the classical nucleation theory to the heterogeneous nucleation of ferrite spherical caps on curved inert inclusion surfaces revealed that the nucleation of ferrite is related to the presence of particles with a larger order of magnitude than 0.1 µm [15]. However, none of the particles larger than 0.1 µm observed in MD and AD steels in this study caused ferrite nucleation.

Moreover, while the surface of the particles is normally an inert interface, it is not the sole one, because austenitic grains grain boundaries are much larger inert interfaces than the particles within the grains. Therefore, it is likely that heterogeneous nucleation alone does not promote particle-excited nucleation and that other factors are required. In this paper, we focus on the discussion of small lattice mismatch and local solute depletion in the AF nucleation mechanism.

#### 4.2.1. Existing Epistemic Relationship between the Nucleant and Nuclei

**Figure 8:**
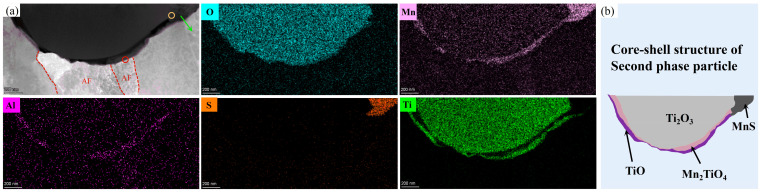
Characterization of the typical core–shell structured particle in TD steel: (**a**) HAADF image with the corresponding EDS elemental maps and (**b**) sketch of the core–shell structure of the second-phase particle.

**Figure 9:**
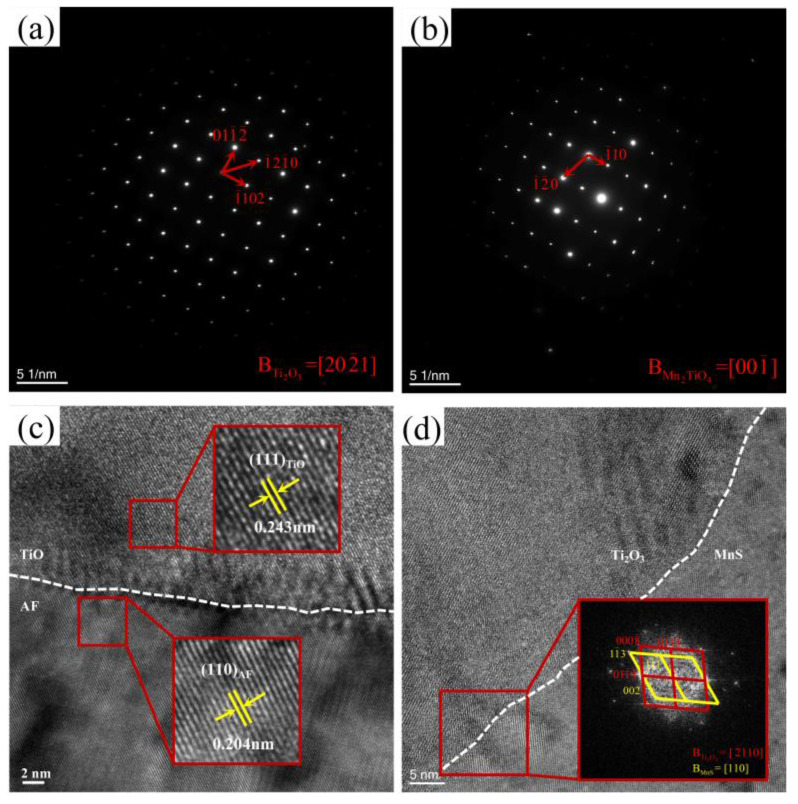
TEM micrographs of the particle obtained in TD steel: (**a**) SAED pattern of Ti_2_O_3_ phase taken along the [21,22] zone axis; (**b**) SAED pattern of Mn_2_TiO_4_ phase taken along the [00-1] zone axis; (**c**) HR-TEM image showing the red circled TiO/AF plate interface in Figure 8a. (**d**) HR-TEM image showing the Ti_2_O_3_/MnS interface indicated by the yellow circle in Figure 8a, inset is the inverse fast Fourier transform (IFFT) pattern of the Ti_2_O_3_/MnS interface.

To further characterize the second-phase particle structure in TD steel, HR-TEM and STEM-EDS are complementarily performed in Figure 8. We can see from the elemental mapping analysis of specific particles (Figure 8a) that the particles have a clear multilayered core–shell structure, in which Ti and O are enriched in the core (containing a little quantity of Mn), and the outer part of the core is wrapped around the (Mn-Ti-O)-rich layer and the (Ti-O)-rich layer; there is also a partial precipitation of MnS on the surface. The Fast Fourier Transform (FFT) plot in Figure 9a confirms that the core is a Ti_2_O_3_ phase. Since the r(Mn^3+^) is similar to the r(Ti^3+^) and there are cationic vacancies within Ti_2_O_3_, Mn atoms are absorbed by Ti_2_O_3_ at high temperatures, resulting in a small amount of Mn being present in the core. The FFT pattern of Figure 9b and the HRTEM image of Figure 9c indicate that the shell structures are Mn_2_TiO_4_ and TiO, respectively. The core–shell structures of the obtained second-phase particles are schematically shown in Figure 8b.

To illustrate the lattice mismatch mechanism of AF nucleation, we examined the orientation relationship of the included TiO/AF plate interface (red circle in Figure 8a), as illustrated in Figure 9c. The AF plate had the following specific orientation with TiO:

(111)_TiO_||(110)_AF_ and [−110]_TiO_||[010]_AF_.

**Figure 10:**
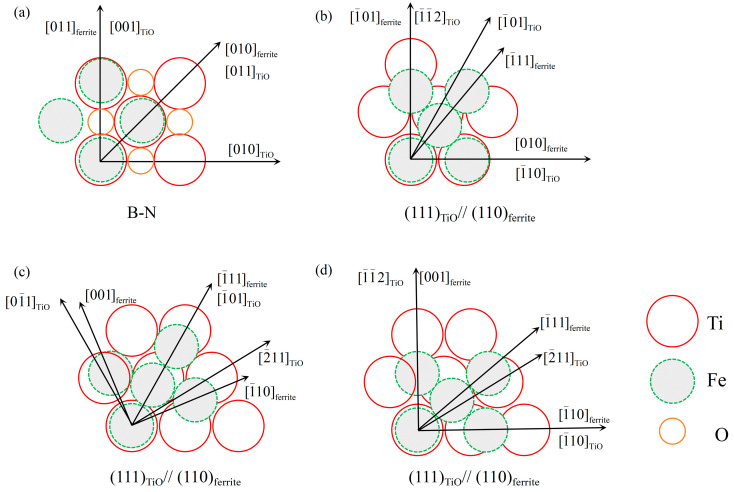
The crystallographic relationship at the interface of TiO/ferrite. (**a**) B-N; (**b**–**d**) (111)_TiO_||(110)_ferrite_.

Previously, Yamada et al. [28] concluded that the interfacial energy between the second-phase particles and the ferrite is lowered in low-carbon submerged arc weld metals due to the (Ti-O)-enriched layer that is present in the amorphous phase and on the surface of MnAl_2_O_4_ oxides. T. Koseki et al. [29] also found that the TiO phase significantly increases the volume fraction of AF, as well as that the epitaxial interaction between ferrite and TiO dominated nucleation. Additionally, it has been demonstrated that B1 (NaCl-type) compounds, like VN, TiN, and TiO, can have a Baker–Nutting (B-N) orientation with ferrite [12,13,30], i.e., {100}_B1_||{100}_α_, <100>_B1_||<110>_α_. This specific orientation relationship realizes a good coherent lattice match between ferrite and B1 compounds. The two-dimensional planar disregistry equation developed by Bramfitt can be used to compute the lattice disregistry between ferrite and the second-phase particle [31]:

(1) $$\delta_{{\left(hkl\right)}_{n}}^{{\left(hkl\right)}_{s}}=\sum_{i=1}^{3}\frac{{|d}_{{\left[uvw\right]}_{s}^{i}}cos\theta_{1}-d_{{\left[uvw\right]}_{n}^{i}}|}{3d_{{\left[uvw\right]}_{n}^{i}}}\times 100\%$$

where (*hkl*)*_n_* represents the low-index crystallographic plane of the new phase; (*hkl*)*_s_* represents the low-index crystallographic plane of the core substrate; [*uvw*]*_n_* represents a low-index crystallographic direction on the crystallographic plane (*hkl*)*_n_*, while [*uvw*]*_s_* represents a low-index crystallographic direction on the crystallographic plane (*hkl*)*_s_*; d[*uvw*]*_n_* represents the atomic spacing in the crystallographic direction [*uvw*]*_n_*; d[*uvw*]*_s_* represents the atomic spacing in the crystallographic direction [*uvw*]*_s_*; and $\theta_{1}$ represents the angle between crystallographic directions [*uvw*]*_s_* and [*uvw*]*_n_*. Table 3 indicates that the lattice parameters of ferrite and TiO were 2.886 Å and 4.293 Å, respectively. Based on the crystallographic relationship (Figure 10a), the lattice disregistry of TiO and ferrite was calculated using Equation (1) and found to be 3% when maintaining the B-N orientation relationship. Meanwhile, we took three possible low-index crystallographic orientations of TiO on the (111) crystal plane. Figure 10b–d depict the crystallographic relationships between TiO and ferrite. The calculated lattice disregistry values between TiO and ferrite were 18.2%, 17%, and 42%, respectively. AF can theoretically nucleate on TiO when ferrite and TiO have a B-N orientation relationship, as demonstrated by Bramfitt [31], who demonstrated that the substrate efficiently supports the nucleation of the nucleating phase when the lattice disregistry between the two phases is less than 12%. The value of the lattice disregistry between (110) of ferrite and (111) of TiO is obviously beyond the range of values required to promote nucleation, so the (111) plane has often been neglected in the selection of the low-index plane of TiO in previous studies. In fact, the results shown in Figure 9c suggest that the lattice structure between the AF plate and TiO was considered to be a semi-coherent interface with a lattice mismatch of about 0.16 based on their measured interplanar spacings of (111)_TiO_ and (110)_AF_ plane (TiO: 0.241 nm, AF: 0.204 nm). For ease of calculation, we assume that the AF nucleates in the planar surface of the second-phase particle, and the activation energy $∆G^{*}$ for AF nucleation is described as follows:

(2) $$∆G^{*}=\frac{16\pi \sigma_{\alpha \gamma }^{3}}{3{\left(∆G_{v}+∆G_{s}\right)}^{2}}\cdot f\left(\theta_{2}\right)$$

(3) $$f\left(\theta_{2}\right)=\frac{\left(2+\cos\theta_{2}\right){\left(1-\cos\theta_{2}\right)}^{2}}{4}$$

(4) $$\cos\theta_{2}=\frac{\left(\sigma_{\gamma p}-\sigma_{\alpha p}\right)}{\sigma_{\alpha \gamma }}$$

where $\sigma_{\alpha \gamma }$ denotes the interfacial energy of AF/austenite phase, $∆G_{v}$ represents the driving force of the transformation, $∆G_{s}$ represents the strain energy, $f\left(\theta_{2}\right)$ represents the shape factor, $\theta_{2}$ represents the contact angle of the nucleus with respect to the precipitation interface, $\sigma_{\gamma p}$ represents the interfacial energy of the austenite/particle, and $\sigma_{\alpha p}$ represents the interfacial energy of the AF/particle. According to Equations (2)–(4), a lower $\sigma_{\alpha p}$ can decrease the $∆G^{*}$. Although the atomic arrangements of (110) for AF and (111) for TiO were different, they had a good semi-coherent lattice match, creating a low-energy interface ($\sigma_{\alpha p}$), therefore causing the interfacial energy and strain energy for the nucleation and the growth of AF on TiO to decrease.

In addition, since the value of 2^−1/2^ times the lattice constant of 4.29 Å for the TiO phase is closer to that of 2.88 Å for the ferrite phase, it is likely that the [010] direction of the ferrite and the [−110] direction of the TiO phase are spatially fairly well matched. The degree of fit of this orientation relationship on the (111)_TiO_||(110)_AF_ surface can be seen in Figure 10b.

#### 4.2.2. MDZ around MnS Precipitates on Ti Oxide Particles

**Figure 11:**
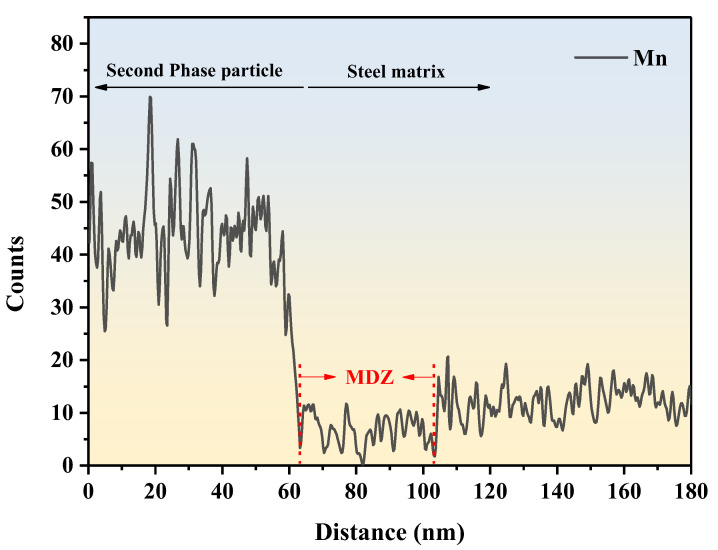
MDZ around the MnS precipitated on Ti oxide in TD steel.

Given that the MDZ mechanism likely significantly affects the development of AF, we analyzed the Mn content close to MnS; the exact position of the line is shown by the green arrow in Figure 8a. Figure 11 shows the variation in Mn elemental content around the MnS precipitated from the dependent second-phase particles. The results show that the Mn concentration decreased as it approached the interface between MnS and ferrite, and there was a clear MDZ near the interface with a width of about 40 nm. Mn depletion impacts IGF nucleation behavior through two mechanisms: austenite supercooling and the ferrite phase transition driving force. As Mn is a γ-forming element, a 1 wt% reduction in Mn concentration in steel causes an approximately 50 °C rise in the γ→α transition temperature [32]. According to Mabuchi et al.’s thermodynamic calculations, the removal of 1.5 wt% of Mn content raised the driving force for ferrite transition by roughly 100 J/mol, in turn encouraging AF nucleation [33].

In addition, Figure 9d shows the HRTEM images and selected FFTs at the interface between Ti_2_O_3_ and MnS, and the results show that the (01-1-4) crystal plane of Ti_2_O_3_ was parallel to the (002) crystal plane of MnS. The orientation relationship that MnS adopted with Ti_2_O_3_ is as follows:${\left(01-1-4\right)}_{{\mathrm{Ti}}_{2}O_{3}}$||(002)_MnS_, ${\left[-2110\right]}_{{\mathrm{Ti}}_{2}O_{3}}$||[110]_MnS_. Since MnS precipitates at a low temperature and readily redissolves into the matrix during heating, this relationship allows the redissolved Mn and S elements to nucleate on high-melting-point Ti_2_O_3_ during the subsequent cooling phase. Wan et al. [34,35] calculated that the cationic vacancy formation energy (0.029 Ry) of Ti_2_O_3_ was smaller than its anionic vacancy formation energy (0.045 Ry). Since the radius of Ti^3+^ is close to that of Mn^3+^, the Mn atoms around Ti_2_O_3_ are adsorbed into its internal cationic vacancy, making it the preferred site for MnS phase precipitation. Since MnS had a particular orientation with Ti_2_O_3_, it is probable that crystallographic variables also impacted MnS precipitation on Ti_2_O_3_, in addition to the precipitation sites that Ti_2_O_3_ provided. The combined effect of these two factors caused MnS to preferentially precipitate at Ti_2_O_3_ attachment, leading to the depletion of the nearby austenite-stabilizing element Mn and promoting AF nucleation.

## 5. Conclusions

This study investigated and compared the mechanical properties and microstructures of hot-rolled mild steels deoxidized with Ti to those of Mn and Al. The correlation between microstructure and impact toughness was investigated, and the mechanisms through which Ti-containing core–shell second-phase particles trigger AF nucleation was clarified. The following are the primary conclusions.

(1) Compared with Al and Mn deoxidized steels, the impact toughness of Ti deoxidized steel at −20 °C was significantly increased by nearly 130 J. The introduced Ti-containing second-phase particles can more effectively promote AF transformation, which contributes to microstructural refinement, and interlocking AF plates with high angular grain boundaries effectively improve the toughness.
(2) TiO, the outermost layer of the second phase containing Ti core–shell particles, can effectively promote the nucleation of AF. TiO has a good semi-coherent relationship with AF and reduces the lattice mismatch, thus effectively stimulating the nucleation of AF.
(3) The MnS precipitation on the Ti_2_O_3_ core adopts a particular orientation relationship of ${\left(01-1-4\right)}_{{\mathrm{Ti}}_{2}O_{3}}$||(002)_MnS_, ${\left[-2110\right]}_{{\mathrm{Ti}}_{2}O_{3}}$||[110]_MnS_, promoting the formation of MDZ near MnS.

Microstructure refinement by particle-stimulated AF nucleation results in superior strength and toughness matching for steel in the hot-rolled state, providing feasible ideas on the microstructure regulation difficulties of thick gauge steel plates under high-temperature hot rolling. However, some bottlenecks still need to be overcome, such as the alloy-joining system’s control mechanism and high-melting-point “beneficial” inclusions in the fine dispersion distribution of the regulatory mechanism; we will continue to investigate these issues in depth in the future.

## Figures and Tables

**Table 1:** Compositions of steels in this experiment (wt%).

Steel	C	Si	Mn	P	S	Cr	Al	Ti
MD	0.12	0.01	1.66	0.015	0.006	0.31	0.003	-
AD	0.13	0.03	1.66	0.016	0.005	0.32	0.02	-
TD	0.12	0.01	1.55	0.015	0.006	0.30	0.002	0.01

**Table 2:** Mechanical properties of the MD, AD, and TD steels.

Steel	Yield Strength [MPa]	Ultimate Tensile Strength [MPa]	Impact Toughness [J]
MD	293 ± 1	465 ± 2	18 ± 2
AD	408 ± 0	587 ± 2	9 ± 2
TD	425 ± 3	584 ± 1	144 ± 16

**Table 3:** Lattice parameters as well as misfits between TiO and ferrite in this study.

Phase	Lattice Parameter [Å]	Parallelisms	Misfit with Ferrite [%]
TiO	4.293	B-N	3
		(111)_TiO_||(110)_ferrite_ [−110]_TiO_||[010]_ferrite_	18.2
		(111)_TiO_||(110)_ferrite_ [−101]_TiO_||[−111]_ferrite_	17
		(111)_TiO_||(110)_ferrite_ [−110]_TiO_||[−110]_ferrite_	42
Ferrite	2.886	-	-

## Data Availability

The original contributions presented in the study are included in the article, further inquiries can be directed to the corresponding authors.

## Abbreviations

**Abbreviation/Symbol**	**Comment**
AF	Acicular ferrite
MDZ	Mn-depleted zone
TMCP	Thermo-mechanical control process
IAF	Intragranular acicular ferrite
PSN	Particle-stimulated nucleation
MDZs	Mn-depleted zones
MD	Mn deoxidized steel
AD	Al deoxidized steel
TD	Ti deoxidized steel
OM	Optical microscope
SEM	Scanning electron microscope
EBSD	Electron backscatter diffraction
STEM	Scanning transmission electron microscopy
FIB	Focused ion beam
HRTEM	High-resolution electron microscopy
SAED	Selected area electron diffraction
B	bainite
PF	Polygonal ferrite
P	Pearlite
FSP	Ferrite side plate
AEGS	Average effective grain size	
LAGBs	Low-angle grain boundaries	
HAGBs	High-angle grain boundaries	
FFT	Fast Fourier transform	
IFFT	Inverse fast Fourier transform	
${\left(hkl\right)}_{n}$	Low-index crystallographic plane of the new phase	
${\left(hkl\right)}_{s}$	Low-index crystallographic plane of the core substrate	
${\left[uvw\right]}_{n}$	Low-index crystallographic direction on the crystallographic plane (*hkl*)*_n_*	
${\left[uvw\right]}_{s}$	Low-index crystallographic direction on the crystallographic plane (*hkl*)*_s_*	
$d{\left[uvw\right]}_{n}$	Atomic spacing along the crystallographic direction [*uvw*]*_n_*	
${d[uvw]}_{s}$	Atomic spacing along the crystallographic direction [*uvw*]*_s_*	
$\theta_{1}$	Angle between crystallographic directions [*uvw*]*_s_* and [*uvw*]*_n_*	
$∆G^{*}$	Activation energy	
$\sigma_{\alpha \gamma }$	Interfacial energy of AF/austenite phase	
$∆G_{v}$	Driving force of the transformation	
$∆G_{s}$	Strain energy	
$f(\theta_{2})$	Shape factor	
$\theta_{2}$	Contact angle of nucleus with respect to the precipitation interface	
$\sigma_{\alpha p}$	Interfacial energy of AF/particle	
$\sigma_{\gamma p}$	Interfacial energy of austenite phase/particle	

## References

[1] Al Hajeri K.F., Garcia C.I., Hua M., Deardo A.J. (2006). Particle-stimulated nucleation of ferrite in heavy steel sections. ISIJ Int..

[2] Sarma D.S., Karasev A.V., Jönsson P.G. (2009). On the role of non-metallic inclusions in the nucleation of acicular ferrite in steels. ISIJ Int..

[3] Wang X., Wang C., Kang J., Yuan G., Misra R.D.K., Wang G.D. (2020). Improved toughness of double-pass welding heat affected zone by fine Ti-Ca oxide inclusions for high-strength low-alloy steel. Mater. Sci. Eng. A.

[4] Wang X., Wang C., Kang J., Wang G.D., Misra D., Yuan G. (2020). Relationship between impact toughness and microstructure for as-rolled and simulated HAZ of low-carbon steel containing Ti-Ca oxide particles. Metall. Trans. A.

[5] Babu S.S., Bhadeshia H.K.D.H. (2007). Mechanism of the transition from bainite to acicular ferrite. Mater. Trans..

[6] Bhadeshia H.K.D.H., Christian J.W. (1990). Bainite in steels. Metall. Trans. A.

[7] Babu S.S., Bhadeshia H.K.D.H. (1990). Transition from bainite to acicular ferrite in reheated Fe-Cr-C weld deposits. Mater. Sci. Technol..

[8] Thewlis G. (1994). Transformation kinetics of ferrous weld metals. Mater. Sci. Technol..

[9] Wang X.M., He X.L. (2002). Effect of boron addition on structure and properties of low carbon bainitic steels. ISIJ Int..

[10] Morral J.E., Cameron T.B. (1977). A model for ferrite nucleation applied to boron hardenability. Metall. Trans. A.

[11] Mu W.Z., Jönsson P.G., Nakajima K. (2017). Recent aspects on the effect of inclusion characteristics on the intragranular ferrite formation in low alloy steels: A review. High. Temp. Mat. Process..

[12] Lee C., Nambu S., Inoue J., Koseki T. (2011). Ferrite formation behaviors from B1 compounds in steels. ISIJ Int..

[13] Takada A., Komizo Y.I., Terasaki H., Yokota T., Oi K., Yasuda K. (2014). Crystallographic analysis for acicular ferrite formation in low carbon steel weld metals. Weld. Int..

[14] Shim J.H., Cho Y.W., Chung S.H., Shim J.D., Lee D.N. (1999). Nucleation of intragranular ferrite at Ti_2_O_3_ particle in low carbon steel. Acta Mater..

[15] Shim J.H., Oh Y.J., Suh J.Y., Cho Y.W., Shim J.D., Byun J.S., Lee D.N. (2001). Ferrite nucleation potency of non-metallic inclusions in medium carbon steels. Acta Mater..

[16] Shim J.H., Byun J.S., Cho Y.W., Oh Y.J., Shim J.D., Lee D.N. (2001). Mn absorption characteristics of Ti_2_O_3_ inclusions in low carbon steels. Scripta Mater..

[17] Gregg J.M., Bhadeshia H. (1994). Titanium-rich mineral phases and the nucleation of bainite. Metall. Trans. A.

[18] (2012). Standard Test Methods for Notched Bar Impact Testing of Metallic Materials.

[19] Dowling J.M., Corbett J.M., Kerr H.W. (1986). Inclusion phases and the nucleation of acicular ferrite in submerged arc welds in high strength low alloy steels. Metall. Trans. A.

[20] Yang Z., Wang F., Wang S., Song B. (2008). Intragranular ferrite formation mechanism and mechanical properties of non-quenched-and-tempered medium carbon steels. Steel Res. Int..

[21] Li X., Min Y., Liu C., Jiang M. (2016). Study on the formation of intragranular acicular ferrite in a Zr-Mg-Al deoxidized low carbon steel. Steel Res. Int..

[22] Liu F., Li J., Wang Q., Liu Y., Bai Y., He T., Yuan G. (2021). Microstructural refinement by the formation of acicular ferrite on Ti-Mg oxide inclusion in low-carbon steel. Mater. Sci. Eng. A.

[23] Curry D.A. (1978). Grain-size dependence of cleavage fracture toughness in mild steel. Nature.

[24] Barritte G.S., Edmonds D.V. (1982). Conference Record of Advances in the Physical Metallurgy and Applications of Steel.

[25] Zhang S., Hattori N., Enomoto M., Tarui T. (1996). Ferrite nucleation at ceramic/austenite interfaces. ISIJ Int..

[26] Zhang Z., Farrar R.A. (1996). Role of non-metallic inclusions in formation of acicular ferrite in low alloy weld metals. Mater. Sci. Technol..

[27] Ricks R.A., Howell P.R., Barritte G.S. (1982). The nature of acicular ferrite in HSLA steel weld metals. J. Mater. Sci..

[28] Yamada T., Terasaki H., Komizo Y. (2008). Microscopic observation of inclusions contributing to formation of acicular ferrite in steel weld metal. Sci. Technol. Weld. Join..

[29] Koseki T., Thewlis G. (2005). Overview Inclusion assisted microstructure control in C-Mn and low alloy steel welds. Mater. Sci. Technol..

[30] Garcia-Mateo C., Capdevila C., Caballero F.G., Andrés C.G.D. (2008). Influence of V precipitates on acicular ferrite transformation part 1: The role of nitrogen. ISIJ Int..

[31] Bramfitt B.L. (1970). The effect of carbide and nitride additions on the heterogeneous nucleation behavior of liquid iron. Metal. Trans..

[32] Shigesato G., Sugiyama M., Aihara S., Uemori R., Tomita Y. (2001). Effect of Mn depletion on intra-granular ferrite transformation in heat affected zone of welding in low alloy steel. Tetsu Hagané.

[33] Mabuchi H., Uemori R., Fujioka M. (1996). The role of Mn depletion in intra-granular ferrite transformation in the heat affected zone of welded joints with large heat input in structural steels. ISIJ Int..

[34] Wan X.L., Wu K.M., Nune K.C., Li Y., Cheng L. (2012). In situ observation of acicular ferrite formation and grain refinement in simulated heat affected zone of high strength low alloy steel. Sci. Technol. Weld. Join..

[35] Wan X.L., Wang H.H., Cheng L., Wu K.M. (2012). The formation mechanisms of interlocked microstructures in low-carbon high-strength steel weld metals. Mater. Charact..

